# Research on image recognition of tomato leaf diseases based on improved AlexNet model

**DOI:** 10.1016/j.heliyon.2024.e33555

**Published:** 2024-06-24

**Authors:** Jing Qiu, Xiaolei Lu, Xinxin Wang, Chen Chen, YouQing Chen, Yi Yang

**Affiliations:** a Teaching Affairs Office, Yunnan Agriculture University, Kunming, 650201, China; b School of Information and Intelligent Engineering, Yunnan College of Business and Management, Kunming, 650106, China; c The Key Laboratory of Crop Production and Smart Agriculture of Yunnan Province, Kunming 650201, China; d College of Big Data(College of Information Engineering), Yunnan Agriculture University, Kunming, 650201, China; e Faculty of Mechanical and Electrical Engineering, Yunnan Agriculture University, Kunming, 650201, China

**Keywords:** AlexNet model, Tomato leaf diseases, Weighted fusion of LBP and HOG, Feature extraction, Recognition model

## Abstract

Aiming at the problems that the traditional image recognition technology is challenging to extract useful features and the recognition time is extended; the AlexNet model is improved to improve the effect of image classification and recognition. This study focuses on 8 types of tomato leaf diseases and healthy leaves. By using HOG and LBP weighted fusion to extract image features, a tomato leaf disease recognition model based on the AlexNet model is proposed, and transfer learning is used to train the AlexNet model. Transfer the knowledge learned by the AlexNet model on the PlantVillage image dataset to this model while reducing the number of fully connected layers. Keras deep learning framework and programming language Python were used. The model was implemented, and the classification and identification of tomato leaf diseases were carried out. The recognition rate of feature-weighted fusion classification is higher than that of serial and parallel methods, and the recognition time is the shortest. When the weight coefficient ratio of HOG and LBP is 3:7, the image recognition rate is the highest, and its value is 97.2 %. From the model performance curve See, when the number of iterations is more than 150 times, the training set and test accuracy rate both exceed 97 %, the loss rate shows a gradient decline, and the change is relatively stable; compared with the traditional AlexNet model, HOG + LBP + SVM model, and VGG model, improved AlexNet model has the highest recognition rate, and it has high recall value, accuracy, and F1 value; Compared with the latest convolutional neural network disease recognition models, improved AlexNet model recognition accuracy was 98.83 %, and the F1 value was 0.994. It shows that the model has good convergence performance, fast prediction speed, and low loss rate and can effectively identify 8 types of tomato leaf images, which provides a reference for the research on crop disease identification.

Tomato has become one of the most important crops in the world because of its rich nutritional value, high yield, easy planting, and other characteristics. In China, tomatoes' planting area and output rank first in the world. According to the Statistical Database of the Food and Agriculture Organization of the United Nations (FAOSTAT2022), in 2021, the world's production of tomatoes was 189.13 million tons, while China's production was 67.53 million tons, accounting for 35.7 % of the world's total production [1]. It is significant to accelerate the development of the agricultural economy, promote farmers' income and improve people's living standards. In recent years, with the expansion of tomato cultivation areas, high yield leading to a single variety, climate change, and other factors, tomato diseases have increased in variety, increased harm, and are more widely distributed, which has seriously affected the yield and quality of tomatoes. It is reported that tomato yield is reduced by more than 30 % annually due to diseases, which may lead to crop failure in severe cases. Therefore, how to effectively identify tomato diseases and take practical measures are of great significance to improve the yield and quality of tomatoes.

The internet of things technology, image recognition technology, and artificial intelligence technology is widely used in various fields. In particular, the successful application of depth learning algorithms in image recognition, especially the convolutional neural network, use convolutional operations to extract important feature information from images, and use pooling operations to compress and average these features, thereby achieving efficient recognition and classification, making intelligent interaction more natural, which provides technical support for improving the accuracy of plant disease recognition. Scholars have used image processing technology to identify plant diseases and have achieved good recognition results. Vijai Singh [2] uses image segmentation technology to detect and classify plant leaf diseases automatically; Srdjan [3] used the deep convolution network to build a plant disease recognition model, and the recognition rate of 13 plant diseases reached 91 %. P. Ganesan [4] used image processing technology to segment effectively and early identifies plant disease spots. Many scholars use deep learning techniques to construct disease recognition models, thereby achieving automatic identification, real-time monitoring, and early warning of crop diseases and pests. Scholars use image processing, such as noise removal, segmentation, and image smoothing threshold method, typical features are extracted, and plant disease recognition models are constructed using convolutional neural networks. They have been applied to recognizing plant diseases, such as apples, peppers, rice, tobacco and vegetables [[5], [6], [7], [8], [9], [10]], with good recognition results.

In recent years, many researchers have made corresponding research on the establishment of tomato disease recognition model and recognition effect, mainly through genetic algorithm, convolution neural networks, and support vector machines to achieve the classification and recognition of plant diseases, and have made specific research achievements. For example, the use of computer vision technology to extract the color, texture, shape, and other features of diseases can effectively improve the automatic recognition ability of conditions [11]; Using a genetic algorithm and BP neural network fusion to establish disease recognition model, the recognition rate of tomato early blight, late blight, and leaf mold reached 92.50 %, 91.25 %, and 95.50 % respectively [12]; The principal component analysis method was used to reduce the dimensions of the sample set, the particle swarm optimization algorithm was used to determine the optimal parameters, and the PCA-SVM disease recognition model was established. The average recognition rate of three tomato diseases was 94.13 % [13]. Fusion of migration learning and convolution neural network to build tomato disease recognition model and achieved good recognition effect [14,15]. Through the construction of the Multi-Scale AlexNet model, image recognition of tomato leaf disease was carried out, making the average recognition accuracy reach 92.7 % [16]; the depth residual network model was improved by replacing part of the standard convolution with depth separable convolution, and the average accuracy of tomato disease identification reached 98.58 % [17]. By constructing a deconvolution-guided VGGNet (DGVGGNet) model to identify disease types and segment disease spots on plant leaves, the disease type recognition accuracy is 99.19 %, the pixel accuracy of disease spot segmentation is 94.66 %, and the average intersection/merge ratio is 75.36 % [18]. Deng et al. (2021) proposed using generative adversarial networks to expand the dataset and evaluated the performance of the test set [19]. Kanda et al. (2022) proposed using residual neural network algorithm to identify tomato diseases, with an F1 score of 99.5 % [20]. Bo et al. (2024) proposed a new Multi Task Distillation Learning (MTDL) framework for the comprehensive diagnosis of tomato leaf disease. The EfficientNet optimized by MTDL only uses 9.46 % of parameters, with better classification accuracy and severity estimation than single task ResNet101 by 0.68 % and 1.52 % [21]. Peng et al. (2023) proposed the Dense Inception MobileNet-V2 parallel convolutional block attention module network (DIMPCNET) for identifying tomato leaf diseases. The results showed that the recognition accuracy and F1 score of DIMPCNET were 94.44 % and 0.9475, respectively, with a loss of approximately 0.28 % [22]. Jiangtao et al. (2022) proposed an improved SE-YOLOv5 network model for identifying tomato virus diseases, with an accuracy of 91.07 % and an average accuracy (mAP (@ 0.5)) of 94.10 % [23]. Wenbo et al. (2023) proposed a sample adaptive cross entropy loss function as a confidence loss, and used MobileNetV3 for lightweight model feature extraction. The improved YOLOX improved the accuracy by 1.27 %, which can better detect tomato leaf disease samples in complex environments [24].

From the current research, tomato disease recognition technology mainly focuses on computer vision technology to construct disease recognition, and has achieved some research results. However, due to the many factors of tomato disease, the same illness has incredible differences in different growth stages, which puts forward higher requirements for image recognition technology. This study plans to use Histogram of Orientated Gradient (HOG) and Local Binary Pattern (LBP) fusion to extract image texture, shape, edge, and other primary features, HOG and LBP fusion feature input to AlexNet network model for extracting advanced image features, using transfer learning to improve models, build a tomato disease recognition model, classify and recognize tomato diseases to provide useful reference and reference for rapid and accurate identification of plant diseases.

## 1 Materials and methods

### 1.1 Datasets

**Fig. 1:**
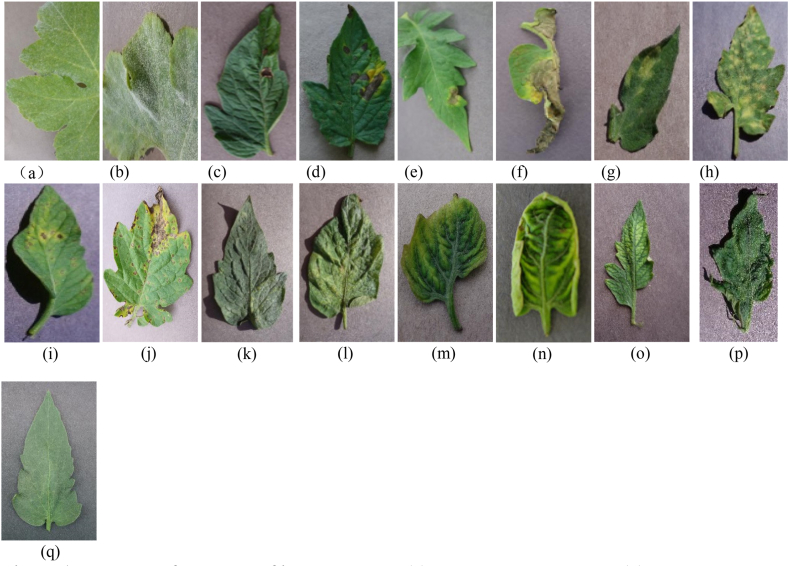
**Example of tomato leaf image dataset. (a)** Moderate powdery mildew; **(b)** severe powdery mildew; (c) moderate early blight; (d) severe early blight; (e) moderate late blight; (f) severe late blight; (g) moderate leaf mold; (h) severe leaf mold; (i) moderate leaf blight; (j) severe leaf blight; (k) moderate red spider damage; (l) severe red spider damage; (m) moderate yellow leaf curl virus disease; (n)severe yellow leaf curl virus disease; (o) moderate mosaic virus; (p)severe mosaic virus; and (q)health.

The tomato leaf images used in this study are the crop disease dataset provided by the 2018 AI Challenger, including eight kinds of diseases such as tomato powdery mildew, early blight, late blight, leaf mold, leaf blight, red spider injury, yellowing leaf curl virus disease, mosaic virus and healthy leaves. Except for healthy leaf, the other seven conditions are divided into moderate and severe according to the degree of illness, with a total of 13038 images; see Table 1 for the number of leaf images of each type. The sample set was imported into the computer in jpeg format to build a tomato disease database. An example of a tomato leaf image is shown in Fig. 1(a) and (b), 1(c), 1(d), 1(e), 1(f), 1(g), 1(h), 1(i), 1(j), 1(k), 1(l), 1(m), 1(n), 1(o), 1(p) and 1(q).

**Table 1:** Summary of datasets used in the study.

Dataset Class	Healthy	Moderate	Severe	Total
Powdery Mildew		365	1104	1469
Early Blight		287	505	792
Late Blight		302	1267	1569
Leaf Blight		481	922	1403
Red Spider Damage		619	310	929
Yellow Leaf Curl virus disease		1616	2826	4442
Leaf Mold		371	384	755
Tomato Mosaic virus		104	194	298
Health	1381			1381
Total	1381	4145	7512	13038

### 1.2 Data processing

**Fig. 2:**
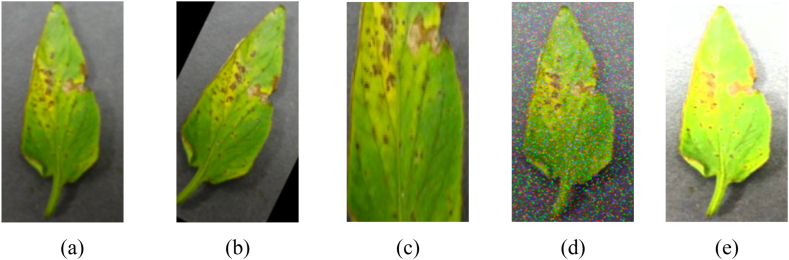
**An Example of the dataset enhancement techniques.**(a) Original image; (b)Rotation; (c) Cutting; (d) Gaussian noise; and (e) Contrast adjustment.

**Fig. 3:**
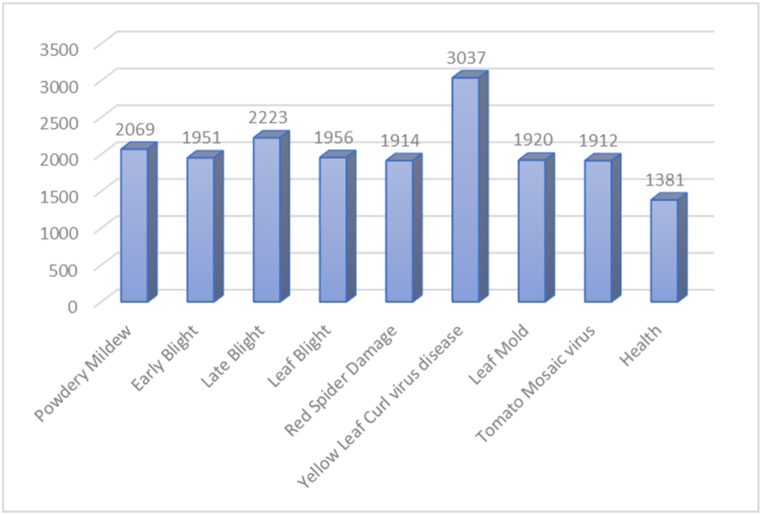
Dataset of this study after data enhancement techniques.

This study uses Python scripting language to find duplicate images and remove them by comparing their names, sizes, and dates. The number of leaves of different categories in the 2018 AI Challenger crop disease dataset tomato leaf dataset needs to be more balanced. To balance the number of images in each category uses, image enhancement techniques such as translation, rotation, cutting, flipping, and perspective transformation are used to expand the samples of the original image. For the categories with too many examples, some models are randomly removed. Each type of sample set is maintained at about 1000, and the total sample set is 18363. The size of all images in the dataset is 256 × 256 pixels. Fig. 2(a) and (b), 2(c), 2(d) and 2(e) show an example image of the enhanced dataset used in this study. Fig. 3 shows the dataset of this study after data enhancement techniques.

### 1.3 Fusion feature extraction

The extraction and selection of image features is the key to image recognition. In this study, HOG and LBP algorithms are used to fuse and extract practical image features to improve the efficiency of image recognition.

#### 1.3.1 HOG feature extraction

HOG is a feature description operator that can well describe the appearance and shape of the local area of the detected object. It reflects the characteristics of the local area of the target image through the gradient size and direction.

(1) HOG feature extraction algorithm
  a. Image standardization

To reduce the difference between local shadow and the illumination of the image and affect the image feature extraction, the input image is grayed, and the gray space is standardized using the gamma correction method.

b. Calculate image gradient

The horizontal and vertical gradients of each pixel in the image are calculated to extract the image contour features. The calculation formula is as follows.

Horizontal gradient of pixel (x, y): $G_{x}\left(x,y\right)=L_{x}\left(x+1,y\right)-L_{x}\left(x-1,y\right)G_{x}\left(x,y\right)=L_{x}\left(x+1,y\right)-L_{x}\left(x-1,y\right)$,

Vertical gradient of pixel (x, y): $G_{y}\left(x,y\right)=L_{y}\left(x,y+1\right)-L_{y}\left(x,y-1\right)$,

Pixel (x, y) gradient amplitude: $G\left(x,y\right)=\sqrt{{G_{x}\left(x,y\right)}^{2}+{G_{y}\left(x,y\right)}^{2}}$,

Pixel (x, y) gradient direction: $\theta \left(x,y\right)=\arctan\frac{G_{y}\left(x,y\right)}{G_{x}\left(x,y\right)}\theta \left(x,y\right)=\arctan\frac{G_{y}\left(x,y\right)}{G_{x}\left(x,y\right)}$.

Gx(x,y)、Gy(x,y)、L(x,y)、G (x,y)、θ(x,y) respectively represent the horizontal gradient, vertical gradient, pixel value, gradient amplitude, and gradient direction at the pixel point (x, y). The value range of the gradient direction is [0,180°].

c. Calculate gradient histogram

Divide the sample images into non-overlapping ones with the same size, 8 × 8 cell units. Divide the gradient direction of pixels into nine groups on average within the 0–180° range. The gradient amplitude corresponding to each pixel in the gradient order of the cell is weighted and projected to obtain the corresponding gradient histogram.

d. Eigenvector normalization

Cell units need to be normalized to improve the effect of image detection and extract useful features. The idea is to combine small cell units into larger blocks, distribute the same cell unit in different blocks to obtain different gradient directions and amplitudes, and then normalize these blocks.

Normalization formula:

$$V\leftarrow \frac{V}{\sqrt{\left(\sum_{k=1}^{n}{\left|V_{k}\right|}^{2}\right)+\varepsilon^{2}}}$$

In the formula, V is a vector, V_k_ is the k-th element of the vector, n is the dimension of the vector, and ε is a very small positive number. It is because avoiding dividing by 0 ensures that the value of V is within a reasonable range, ensures numerical stability, and prevents overfitting; Even if the norm of V is small, the normalized vector still has some non-zero values, thus achieving smoothing processing.

f. Form HOG eigenvector

The HOG feature vectors of all overlapping blocks are integrated to form a 3780-dimensional feature vector for image classification and recognition.

(2) HOG algorithm feature extraction results

**Fig. 4:**
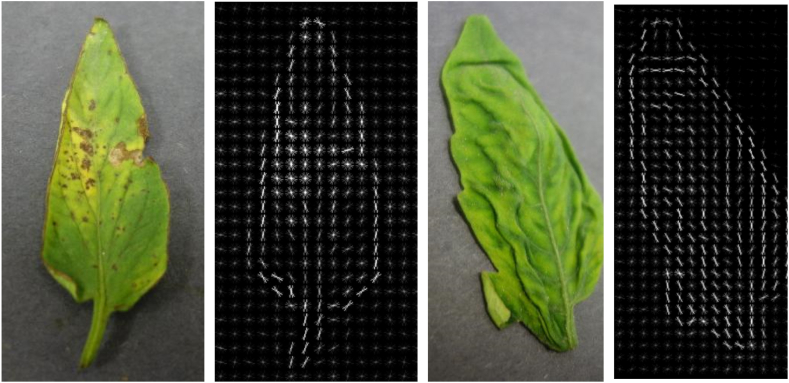
Extracting tomato image features using HOG algorithm.

Use the HOG algorithm to extract the appearance and shape of the image, and the effects of some extracted images are shown in Fig. 4.

#### 1.3.2 LBP feature extraction

LBP is used to extract local texture features of images, with rotation and grayscale invariance. It is mainly used in image recognition, computer vision, image processing.

(1) LBP feature extraction algorithm
  a. Divide Image

Divide the Image into cells of the same size.

b. Calculate the LBP value

The threshold value of each pixel in the cell unit is calculated and compared with the threshold value of the adjacent 8 pixels. If the threshold value of the adjacent pixel is greater than or equal to the threshold value of the central pixel, the value of the adjacent pixel is recorded as 1; otherwise, it is 0. The resulting 8-bit binary number is converted into decimal, which is the LBP value of the central pixel.

c. Calculation histogram

Calculate the histogram of each cell unit, and the histogram projection is the LBP value.

d. Histogram normalization

The histogram of each cell unit is normalized.

f. Form LBP eigenvector

The normalized histogram is connected to a feature vector, the LBP texture feature vector of the Image, for image classification and recognition.

(2) LBP algorithm feature extraction results

**Fig. 5:**
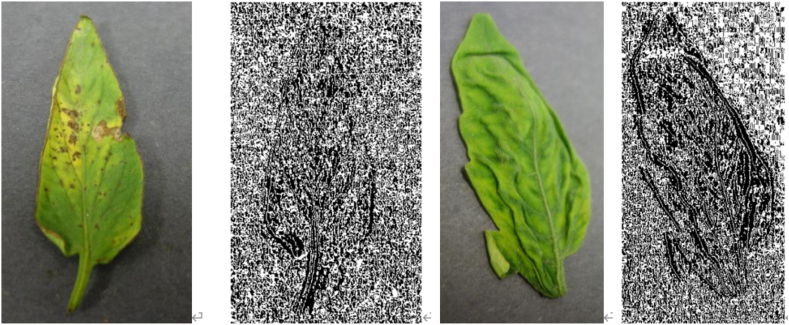
Extracting tomato image features using LBP algorithm.

The LBP algorithm is used to extract the texture features of the Image. The results of removing the texture features of some tomato images are shown in Fig. 5.

#### 1.3.3 Image fusion feature extraction

**Fig. 6:**
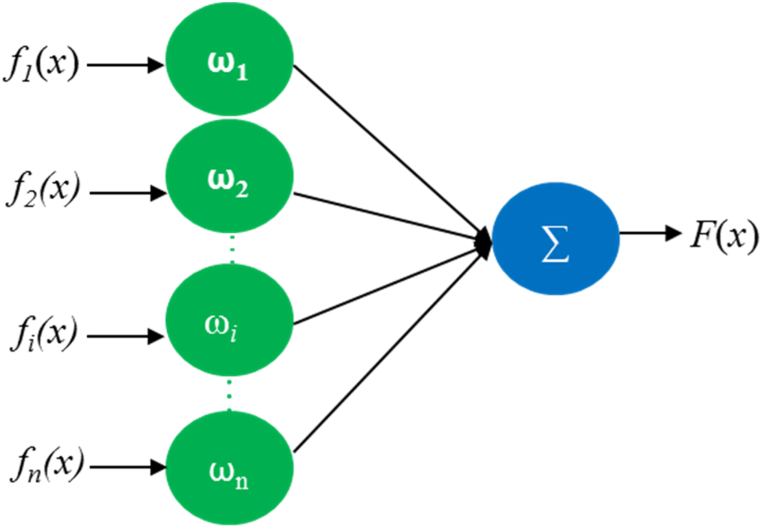
Principle of weighted fusion algorithm.

There are three main methods for feature extraction of image fusion: serial fusion, parallel fusion, and weighted fusion. Serial fusion is connecting two vectors head to tail to form a new feature vector, and the dimension of the new vector feature increases sharply, thus reducing the speed and accuracy of image recognition; Parallel fusion combines two groups of feature vectors into complex feature vectors. The parallel feature vectors formed by the algorithm are biased due to the imbalance problem, which reduces the image recognition effect; Weighted fusion is to assign different weight coefficients according to the additional contributions of additional features in the image recognition process so that the image information is more accurate, the credibility is higher, the signal-to-noise ratio of the image is improved, and the image recognition rate is improved. The principle of weighted fusion algorithm is shown in Fig. 6.

In Fig. 6, $f_{i}\left(x\right)\left(i=1,2,\ldots ,n\right)$ represents the i-th extracted feature, $\omega_{i}$ is the weight occupied by the i-th feature $f_{i}\left(x\right)$, $\sum_{i=1}^{n}\omega_{i}=1$, F (*x*) is the feature vector weighted by n features.

This study uses weighted fusion. To eliminate the dimensional influence between different extracted features, before image feature fusion, the extracted LBP and HOG feature vectors are normalized, and the feature value range is between [0,1].

### 1.4 Disease identification model

Because the selection of network architecture dramatically impacts the accuracy of image classification and recognition, the choice of recognition model architecture is essential. The convolutional neural network is a feedforward neural network, essentially a variety of multilayer perceptron. It can automatically extract image features from images, widely used in image classification, computer vision processing, and other fields.

#### 1.4.1 AlexNet model structure

**Fig. 7:**
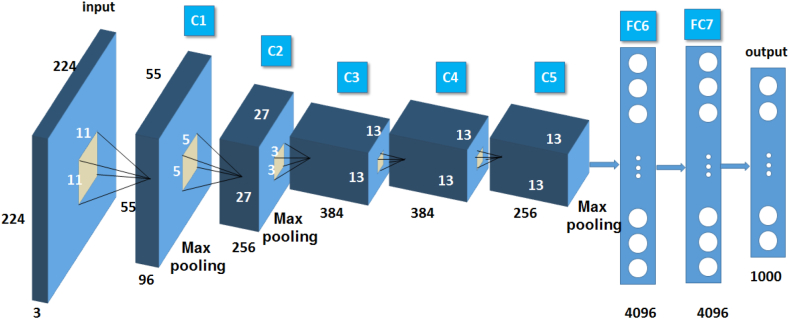
AlexNet model structure.

The AlexNet model, as a classic model of convolutional neural networks, was proposed by Krizhevsky A [25] et al., in 2012 and achieved classification of 1000 image categories. The AlexNet model consists of one input layer, five convolutional layers, three max pooling layers, three fully connected layers, and one output layer. The output layer is a 1000 class Softmax layer. The pooling layer is located after the convolutional layer and uses the activation function ReLu for nonlinear operations. The model parameters are updated through backpropagation algorithm. The activation function of the convolutional layer is ReLu, and the classifier is softmax. Dropout layers are set in Fc6 and fc7 to prevent overfitting of the model. The network structure of AlexNet model is shown in Fig. 7, and the network model parameters are shown in Table 2.

**Table 2:** AlexNet network model parameters.

Network Layer	Input data size	Convolutional layer	Number	Convolutional kernel size	Convolutional step size
Conv 1	224 × 224 × 3	11 × 11	96	4	55 × 55 × 96
Maxpooling	55 × 55 × 96	3 × 3	–	2	27 × 27 × 96
Conv 2	27 × 27 × 96	5 × 5	256	1	27 × 27 × 256
Maxpooling	27 × 27 × 256	3 × 3	–	2	13 × 13 × 256
Conv 3	13 × 13 × 256	3 × 3	384	1	13 × 13 × 384
Conv 4	13 × 13 × 384	3 × 3	384	1	13 × 13 × 384
Conv 5	13 × 13 × 384	3 × 3	256	1	13 × 13 × 256
Maxpooling	13 × 13 × 256	3 × 3	–	2	6 × 6 × 256
Fully connected	6 × 6 × 256	–	4096	–	1 × 4096
Fully connected	1 × 4096	–	4096	–	1 × 4096
Fully connected	1 × 4096	–	1000	–	1 × 1000

#### 1.4.2 Disease recognition model based on transfer learning

The commonly used methods for transfer learning include pre training and fine-tuning. Pre training refers to training a deep neural network model on a large dataset, using pre trained model parameters as the starting point for a new task, and fine-tuning it on the dataset of the new task. Fine tuning usually only involves updating a part of the model's layers (such as fully connected layers), while keeping the parameters of other layers (such as convolutional layers) unchanged or making minor updates.

This study uses the AlexNet model as a pre training model, which can reduce network training parameters, accelerate network convergence, and improve recognition accuracy. Pre train the AlexNet model using the PlantVillage dataset to classify 38 types of plant leaf diseases and healthy leaves, and save the parameters and results of the pre trained model.

**Fig. 8:**
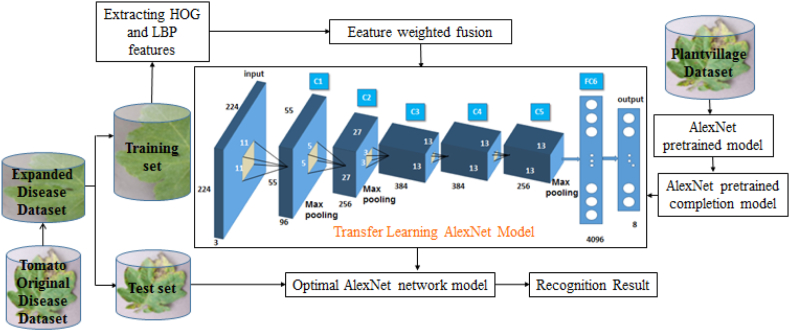
Flowchart of tomato leaf disease recognition model based on transfer learning.

Tis study used transfer learning with structural and parameter fine-tuning to identify tomato leaf diseases. Mainly removing the second fully connected layer from the three fully connected layers in the AlexNet model. Meanwhile, modify the output layer dimension of the AlexNet model. Due to the fact that the disease recognition model classifies and recognizes 8 types of tomato leaf images, the dimension of the output layer is 8, in order to construct a tomato leaf disease recognition model based on transfer learning. The model consists of 7 layers, 5 convolutional layers, and 2 fully connected layers. The DropOut parameter of the 6th fully connected layer is modified to 0.25 to prevent overfitting during training; Still using the soft function to classify disease images, the convolutional layer activation function uses the Relu function, and the loss function is calculated using cross entropy. The process of tomato leaf disease recognition model based on transfer learning is shown in Fig. 8.

## 2 Results and analysis

### 2.1 Experimental environment and setup

Experimental environment: This study used Windows 10 operating system, Intel (R) Xeon (R) CPU E5-2680 v4 @ 2.40 GHz, NVIDIA RTX A2000, 32 GB memory, CUDA 11.0 framework, and CuDNN 8.0 acceleration library. Keras deep learning framework, programming language Python. The experimental environment is shown in Table 3.

**Table 3:** Experimental environment.

Experimental environment configuration	Parameter values Enhancement
Operating System	Ubuntu
Programming Language	Python3.8.5
CPU	E5-2680 v4 @ 2.40 GHz
GPU	NVIDIA RTX A2000
RAM	32
CUDA	11.0
CuDNN	8.0
Pythorch	11.71

This study divided the tomato leaf disease dataset into training set, validation set, and test set. The training set have 16563 images, accounting for 90 % of the dataset, while the test set have 1800 images, accounting for 10 % of the total dataset. The resolution of the input image is 256 × 256, and the image is cropped to 224 × 224 using random cropping. The loss function is cross entropy, and the learning rate is 0.001. The model trains 128 images per batch (batch size), with a training period of 200 cycles (epochs).

### 2.2 Evaluation index

This study comprehensively evaluates the effectiveness of the disease identification model based on indicators such as Accuracy, Recall, Precision, and F1 score of the confusion matrix. The derivation of indicators is as follows.

$$accuracy=\frac{TP+TN}{TP+TN+FP+FN}$$

$$recall=\frac{TP}{TP+FN}$$

$$precision=\frac{TP}{TP+FP}$$

$$F_{1}=\frac{2\cdot recall\cdot precision}{recall+precision}$$

In the formula: Recall: recall rate; F1 is the harmonic mean of the balanced F-score, precision, and recall; TP: true positive; TN: true negative; FP: False positive; FN: False negative.

### 2.3 Comparison experiment of different feature fusion methods

This experiment extracts HOG and LBP features from all images in the test set, and performs serial feature fusion, parallel feature fusion, and weighted feature fusion respectively. The obtained image fusion features are pushed in batches to the already trained AlexNet model for image recognition. The number of images in each batch is 128,it's epochs is 200, and the image resolution is 224 × 224, to verify which fusion feature has better recognition effect on disease images. The experimental results are shown in Table 4.

**Table 4:** Comparison of image recognition rates with different feature fusion methods.

feature fusion method	time (s)	accuracy(%)
serial fusion	16.25	91.2
parallel fusion	14.68	92.1
weighted fusion	13.53	97.2

Table 4 shows that the average recognition rate and recognition time of weighted feature fusion images are 97.2 % and 13.53s, respectively, the average recognition rate of parallel and serial feature fusion images are 92.1 % and 91.2 %, respectively, and the recognition time is 14.68s and 16.25s respectively. The recognition rate of weighted feature fusion images is significantly higher than that of serial and parallel feature fusion images; the image recognition time of weighted feature fusion is less than that of serial and parallel feature fusion. It shows that weighted feature fusion improves the model's image recognition rate and recognition efficiency.

### 2.4 Determination of characteristic weight coefficient

**Fig. 9:**
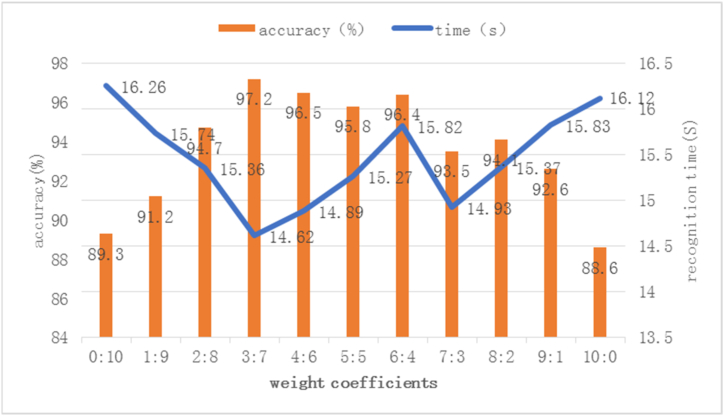
Image recognition time and recognition rate with different weight coefficients.

In this experiment, it's epochs is 200. Image recognition experiments are carried out by setting different weight coefficients of HOG and LBP to determine the best weight coefficient. The experimental results are shown in Fig. 9.

It can be seen from Fig. 9 that when the weight ratio of HOG and LBP is 3:7, the image recognition rate is the highest and the recognition time is the shortest, in which the image recognition rate is 97.2 % and the recognition time is 14.62s; Only the HOG feature is extracted, the image recognition rate is the lowest, and the recognition time is extended. It shows that the image recognition rate of weighted fusion is significantly higher than that of single-feature extraction, and it is optimal when the weight ratio of HOG and LBP is 3:7.

### 2.5 Model performance analysis

**Fig. 10:**
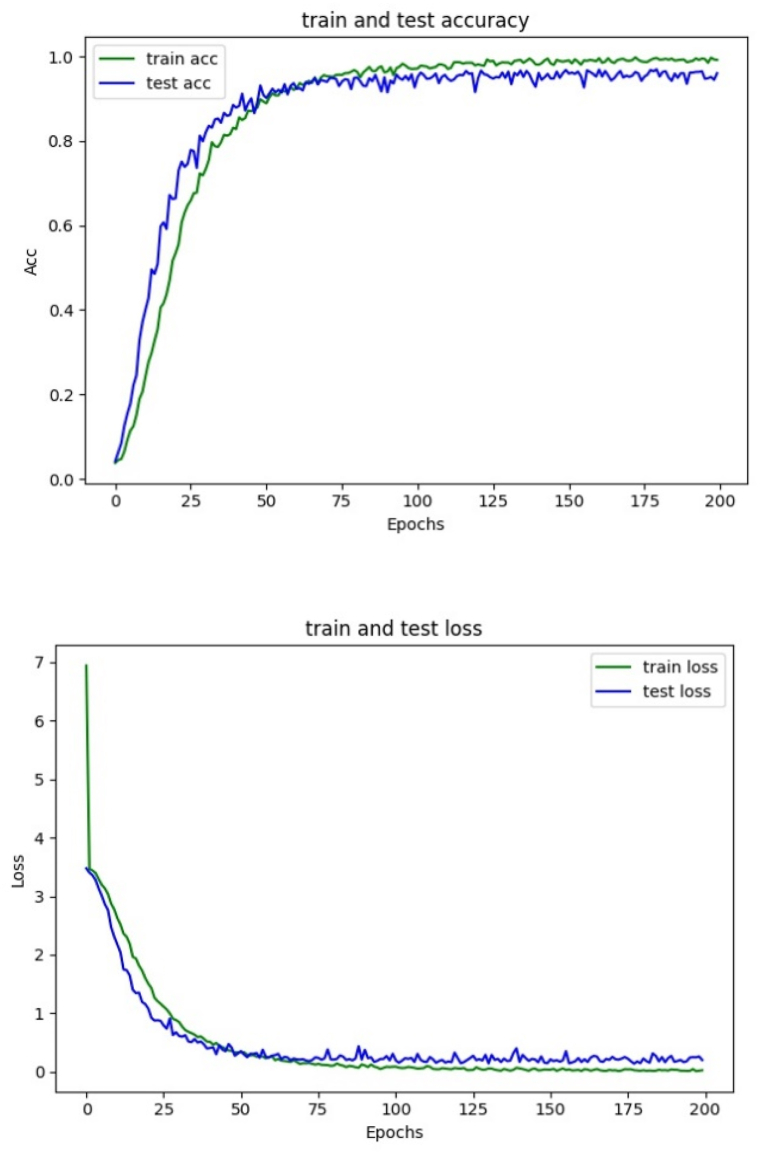
Analysis of the classification and recognition performance of tomato leaf images.

The model was used to classify tomato leaf images, and the convergence of the model was verified. The simulation experiment results are shown in Fig. 10.

It can be seen from Fig. 10 that according to the accuracy curve of the training set and test set, the accuracy of the model training set and test set gradually increases with the increase of iteration times. It shows that it’s epochs >100, the training set and test set accuracy exceeds 90 %. and it's epochs>150, the accuracy curve of the training set and test set tends to be stable, and the accuracy of the training set and test set exceeds 97 %. According to the training and test set's loss rate curve, the curve shows a downward gradient trend. When it's epochs>150, the curve tends to be stable without overfitting, which indicates that the model has strong generalization ability and good convergence performance.

### 2.6 Performance comparison of different models

**Fig. 11:**
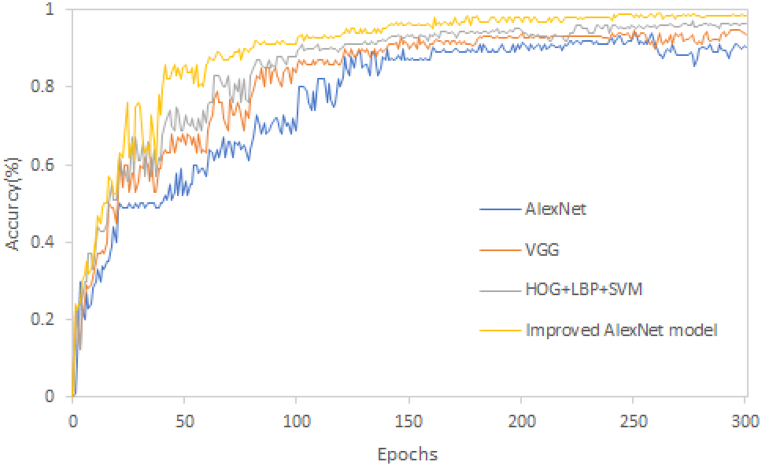
Comparison of image classification and recognition of different models.

To study the image classification and recognition performance of this model and determine its recognition efficiency, the traditional AlexNet model, VGG model, HOG + LBP + SVM model, and the improved AlexNet model were used to carry out image classification and recognition experiments on tomato leaf images. Each experiment used the same experimental environment. The experimental results are shown in Table 5 and Fig. 11.

**Table 5:** Classification accuracy rates of different model diseases.

Model	Accuracy(%)	Recall(%)	Precision(%)	F_1_(%)
AlexNet	87.00	93.24	92.57	92.90
VGG	92.17	97.03	94.72	95.86
HOG + LBP + SVM	91.00	96.45	93.97	95.19
Improved AlexNet model	98.72	98.91	99.77	99.34

Table 5 shows it the improved AlexNet model has the best disease classification and recognition performance, with an accuracy of 98.72 %, and has high recall value, accuracy, and balanced F-score. This indicates that the model improved AlexNet model has good recognition performance and better prediction performance than the other three models.

It can be seen from Fig. 11 that from the perspective of recognition accuracy, the classification accuracy of the improved AlexNet model is higher than that of the other three models, followed by the image recognition accuracy of the HOG + LBP + SVM model, and the traditional AlexNet model is the most miniature ideal model; When the number of iterations is more than 100, the image classification and recognition accuracy of the improved AlexNet model is more than 90 %. When epochs> 140, the image classification and recognition accuracy of the VGG model and the HOG + LBP + SVM model is more than 90 %. When epochs>150, the classification and recognition accuracy of the traditional AlexNet model is more than 90 %; this shows that the improved AlexNet model has the highest image recognition efficiency. From the perspective of image stability, the improved AlexNet model is more stable than the other three models in terms of image stability without fluctuation. In comparison, the other three models have some changes. It shows that the improved AlexNet model has better recognition performance for tomato leaf images and has certain recognition speed and robustness advantages.

### 2.7 Comparison with the latest network models

Using the dataset provided in this study, the improved AlexNet model was compared with classic models such as MobileNetV3, EfficientNet, DenseNet121, and ResNet. In order to investigate the effectiveness of the improved AlexNet model, each experiment was conducted in the same experimental environment. The results are shown in Table 6.

**Table 6:** Accuracy rates of the latest network models.

Model	Accuracy(%)	Recall(%)	Precision(%)	F_1_(%)
MobileNetV3	94.11	95.87	98.01	96.93
EfficientNet	94.78	99.51	95.01	97.21
DenseNet121	95.28	95.51	94.15	94.83
ResNet	91.56	91.55	99.75	95.47
Improved AlexNet model	98.83	99.15	99.66	99.40

Table 6 shows that compared with MobileNetV3, EfficientNet, DenseNet121, and ResNet, the improved model proposed in this study has the highest accuracy of 98.83 %. the improved AlexNet model proposed in this study has the highest accuracy, with an accuracy of 98.83 %, corresponding to improvements of 4.72 %, 4.06 %, 3.56 %, and 7.82 %. The results indicate that the improved AlexNet model outperforms some of the latest crop disease classification models.

### 2.8 Robustness analysis of the model

Using a single test set to train and evaluate the model may have certain randomness, which is not conducive to observing the stability of the model. Therefore, in order to better validate the model, a 10 fold cross validation method was used in the experiment to evaluate the model. The tomato leaf disease dataset will be divided into 10 parts, with 9 parts selected as the training set and 1 part as the testing set. The average of 10 experimental results will be taken as the evaluation standard for model robustness. The experimental results are shown in Table 7.

**Table 7:** Outcome of ten-fold cross-validation method.

Fold Numbers	1	2	3	4	5	6	7	8	9	10	mean value
Training Accuracy (%)	98.81	98.95	99.01	98.81	98.93	98.78	98.91	98.89	98.92	98.85	98.89
Test Accuracy(%)	98.87	98.85	98.91	98.83	98.94	98.81	98.87	98.79	98.97	98.93	98.88

Table 7 shows that after 10 experiments, the maximum training accuracy was 99.1 %, the minimum was 98.81 %, and the average training accuracy was 98.89 %; The maximum test accuracy is 98.94 %, the minimum is 98.81 %, and the average vehicle accuracy is 98.88 %; The difference between the accuracy of 10 experimental training and testing is very small, indicating that the model has a certain degree of stability and reliability.

## 3 Conclusion

Tomato diseases are affected by many factors, and the same illness will have significant differences in different growth stages, leading to poor recognition effect, strong dependence on image features, and poor generalization ability. In this study, the fusion of HOG and LBP is used to extract the primary components of the image, and the AlexNet network model is used to extract advanced image features, build a tomato disease recognition model, and classify and recognize the tomato leaves. The following conclusions are obtained.

1) After serial fusion, parallel fusion, and weighted fusion of the features extracted from HOG and LBP, tomato images are classified and recognized. The experimental results show that the classification accuracy of weighted fusion is higher than that of serial and parallel fusion, and the recognition time is the shortest.
2) The optimal weight is determined by setting the weight coefficients with different characteristics between HOG and LBP. The experiment shows that when the weight coefficient ratio of HOG to LBP is 3:7, the image recognition rate is 97.2 %, and the recognition time is 14.62s. The image recognition rate and recognition time are better than other weight coefficient ratios.
3) From the model performance analysis, the accuracy curves of the training set and test set tend to be stable without fluctuation; with the increase of iterations, the accuracy of the image training set and test set increases gradually. When the iterations are more than 150, the accuracy of training set and test set exceeds 97 %; The loss rate curves of training set and test set show a downward gradient trend, indicating that the model has strong generalization ability and good convergence performance.
4) The model is compared with the traditional AlexNet model, HOG + LBP + SVM model, and VGG model for classification and recognition. It was found that the improved AlexNet model had higher recognition accuracy than the other three models, with a recognition accuracy of 98.72 % and a balanced F-score of 99.4 %. Moreover, it had a higher recall value and accuracy, indicating that the performance of this model is better than the other three models. The improved AlexNet model has improved the average accuracy by comparing the latest convolutional neural network disease recognition models. The results indicate that the improved AlexNet model outperforms some of the latest crop disease classification models. Meanwhile, the results of ten-fold cross-validation indicate that the model has good robustness.

In addition, our proposed disease identification model also has certain limitations. Firstly, the research on the disease recognition model was conducted in experimental environments. The tomato leaf disease images used were all background removed, and more attention was paid to identifying individual individuals in the images. How to classify and recognize images in natural environments, extract effective and typical features of the images, and classify and recognize crop images to improve the practicality and usability of the model. Secondly, currently, we only consider how to effectively identify diseases if there is only one disease on a single leaf. In addition, although we have classified the severity of diseases, we have not paid attention to the development and changes of diseases. How to detect diseases early and take targeted measures in a timely manner. These will be the topics we will focus on in the next step, which will help improve the recognition effect of tomato leaf diseases and provide ideas and references for crop diseases.

## Data availability statement

Data will be made available on request.

## CRediT authorship contribution statement

**Jing Qiu:** Writing – original draft, Formal analysis, Data curation, Conceptualization. **Xiaolei Lu:** Writing – original draft, Software, Methodology, Formal analysis, Data curation. **Xinxin Wang:** Methodology, Formal analysis. **Chen Chen:** Writing - review & editing, visualization, supervision. **YouQing Chen:** Formal analysis, investigation, resources. **Yi Yang:** Writing – review & editing, Visualization, Validation, Supervision, Software.

## Declaration of competing interest

The authors declare that they have no known competing financial interests or personal relationships that could have appeared to influence the work reported in this paper.
